# USP7 facilitates SMAD3 autoregulation to repress cancer progression in p53-deficient lung cancer

**DOI:** 10.1038/s41419-021-04176-8

**Published:** 2021-09-27

**Authors:** Yu-Ting Huang, An-Chieh Cheng, Hui-Chi Tang, Guo-Cheng Huang, Ling Cai, Ta-Hsien Lin, Kou-Juey Wu, Ping-Hui Tseng, Greg G. Wang, Wei-Yi Chen

**Affiliations:** 1 Institute of Biochemistry and Molecular Biology, National Yang Ming Chiao Tung University, Taipei, 112 Taiwan; 2 Department of Life Sciences and Institute of Genome Sciences, National Yang Ming Chiao Tung University, Taipei, 112 Taiwan; 3 Department of Biochemistry and Biophysics, University of North Carolina at Chapel Hill School of Medicine, Chapel Hill, NC USA; 4 Lineberger Comprehensive Cancer Center, University of North Carolina at Chapel Hill School of Medicine, Chapel Hill, NC USA; 5 Basic Research Division, Medical Research Department, Taipei Veterans General Hospital, Taipei, 112 Taiwan; 6 Cancer Genome Research Center, Chang Gung Memorial Hospital at Linkou, Taoyuan, 333 Taiwan; 7 Cancer Progression Research Center, National Yang Ming Chiao Tung University, Taipei, 112 Taiwan

**Keywords:** Proteases, Non-small-cell lung cancer, Growth factor signalling, Transcriptional regulatory elements

## Abstract

USP7, one of the most abundant ubiquitin-specific proteases (USP), plays multifaceted roles in many cellular events, including oncogenic pathways. Accumulated studies have suggested that USP7, through modulating the MDM2/MDMX-p53 pathway, is a promising target for cancer treatment; however, little is known about the function of USP7 in p53-deficient tumors. Here we report that USP7 regulates the autoregulation of SMAD3, a key regulator of transforming growth factor β (TGFβ) signaling, that represses the cell progression of p53-deficient lung cancer. CRISPR/Cas9-mediated inactivation of *USP7* in p53-deficient lung cancer H1299 line resulted in advanced cell proliferation in vitro and in xenograft tumor in vivo. Genome-wide analyses (ChIP-seq and RNA-seq) of *USP7* KO H1299 cells reveal a dramatic reduction of *SMAD3* autoregulation, including decreased gene expression and blunted function of associated super-enhancer (SE). Furthermore, biochemical assays show that SMAD3 is conjugated by mono-ubiquitin, which negatively regulates the DNA-binding function of SMAD3, in *USP7* KO cells. In addition, cell-free and cell-based analyses further demonstrate that the deubiquitinase activity of USP7 mediates the removal of mono-ubiquitin from SMAD3 and facilitates the DNA-binding of SMAD3-SMAD4 dimer at *SMAD3* locus, and thus enhance the autoregulation of *SMAD3*. Collectively, our study identified a novel mechanism by which USP7, through catalyzing the SMAD3 de-monoubiquitination, facilitates the positive autoregulation of *SMAD3*, and represses the cancer progression of p53-deficient lung cancer.

## Introduction

Ubiquitination is a type of posttranslational modification in that ubiquitin is covalently conjugated to target proteins through a process catalyzed by multiple enzymes, including the E1 ubiquitin-activating enzymes, the E2 ubiquitin conjugation enzymes, and the E3 ubiquitin ligases [1]. Different types of ubiquitin modifications have been reported and assigned to distinct functions. For example, the addition of a single (mono-) or non-Lys-48-linked polyubiquitin to protein substrates may serve a non-degradative function, such as trafficking, protein–protein interaction, and modulating the activity of enzymes. On the other hand, the Lys-48 polyubiquitinated proteins are often recognized and subjected to degradative processes mediated by the 26 S proteasome pathway [2, 3]. Conversely, deubiquitination, through the action of deubiquitinating enzymes (DUBs), are responsible for the removal of the ubiquitin/polyubiquitin chain from protein substrates and reverses the functions of ubiquitination [4]. Like most posttranslational modifications, ubiquitination has also been implicated in numerous cellular functions, including cell-cycle progression, apoptosis, gene transcription, DNA repair, and signal transduction that consequently regulate cell development and differentiation, as well as tumorigenesis [5]. Therefore, components in ubiquitin pathways have been proposed as potential targets for therapeutic strategies against diseases and cancers [6].

There are nearly 100 deubiquitinating enzymes that constitute a large superfamily of ubiquitin-proteases encoded by the human genome [4]. Based on the catalytic domains, these DUBs are classified into five subfamilies, including the ubiquitin-specific proteases (USPs), ubiquitin C-terminal hydrolases (UCHs), Machado–Joseph disease proteases (MJDs), and ovarian tumor proteases (OTUs) belong to Clan A of cysteine proteases, and JAB1/MPN/Mov34 (JAMMs) belongs to zinc-dependent metalloproteases [4, 7]. Among these subfamilies, USPs are most abundant and intensively investigated. An increasing number of studies have indicated that several USPs are aberrantly upregulated and promote the formation of various tumors. One of the most well-studied cases is the ubiquitin-specific protease 7 (USP7), also named herpesvirus-associated ubiquitin-specific protease (HAUSP), which plays multifaceted roles involved in viral [8] and oncogenic [9] pathways [10]. USP7 has a broad range of substrates, including p53 tumor suppressor and its negative regulators Murine double minutes (MDM2)/Murine double minutes X (MDMX) [11], and it is proposed that the oncogenic activity of USP7 is through modulating the balance of MDM2/MDMX-p53 circuity. Targeting or inactivating USP7 was reported to destabilize the MDM2/MDMX and stabilize/activate the p53 function that leads to tumor suppression [12]. Therefore, accumulated studies have suggested that USP7 is a promising target for cancer therapy [10, 13] and thus several small-molecule compounds, such as HBX 41,108 [14], and P5091 [15], has been developed to inhibit the enzymatic activity of USP7 and showed promising antitumor functions in various cancer models. Beyond these studies, there has been no comprehensive analyses of the cellular function of USP7 in p53-deficient cancer cells.

The transforming growth factor-β (TGF-β)/SMAD signaling is also a multifunctional pathway involved in several biological functions, including embryogenesis, cell development, and differentiation [16–22]. In addition, TGF-β/SMAD signaling has also been reported to act as tumor-promoting and tumor suppressing pathways in early-stage and advanced cancers, respectively [23–26]. The TGF-β receptors, which endow intrinsic serine/threonine kinase activity, are activated by interaction with specific dimeric ligands and subsequently phosphorylate the receptor-associated SMADs (R-SMADs), such as SMAD2 and SMAD3, to activate the downstream signaling [27]. The phosphorylated R-SMADs can assemble a heterodimer with cooperating SMADs (Co-SMADs), such as SMAD4 [28, 29], and translocate into the nucleus to regulate downstream target genes. In addition to phosphorylation, the R-SMADs are also regulated by other posttranslational modifications. For example, the SMAD3 was reported to be conjugated with mono-ubiquitin [30] that inhibits the formation of homotrimeric SMAD3 or heterotrimeric complex with SMAD4, and thus impairs the DNA-binding function of SMAD3.

In this study, *USP7* was inactivated by CRISPR/Cas9-mediated gene editing in p53-null lung cancer H1299 cells. Unexpectedly, the *USP7* KO cells displayed an advanced cell proliferation and tumor growth in the xenograft murine model. Genome-wide analyses further identified that USP7 is specifically required for the transcriptional activation of *SMAD3*, which is involved in repressing cancer cell proliferation. Finally, our biochemical analyses established that the deubiquitinase function of USP7 can mediate the removal of mono-ubiquitin from SMAD3 and, thus, facilitate the positive autoregulation of SMAD3. Collectively, our findings uncovered a previously unappreciated function for USP7 in regulating the *SMAD3* autoregulation and repressing the cell proliferation of p53-deficient cancer cells.

## Materials and methods

### Cell culture

Human lung cancer H1299, embryonic kidney 293 T (HEK293T), HEK293 cells were grown in Dulbecco modified Eagle’s medium (DMEM, Hyclone); human lung cancer A549 cells were grown in RPMI 1640 medium (Hyclone). Culture media were supplemented with 10% fetal bovine serum (FBS, Invitrogen) and 1% penicillin/streptomycin (P/S, Invitrogen). Cells were maintained at 37 °C in 5% CO_2_ and 95% humidity. Insect High Five and Sf9 cells were cultured in Grace’s insect medium (Invitrogen) supplemented with 10% FBS, 50 μg/ml gentamycin, and 0.1% Poloxamer 188 solution (Sigma).

### Immunoblotting and antibodies

For immunoblotting assays, the indicated cells were counted after trypsinization and directly lysed in Laemmli sample buffer. The cell lysates equivalent to 50,000 cells were separated by gel electrophoresis and immunoblotted with the indicated antibodies. Antibodies used in this study were anti-USP7 (Bethyl, A300-033A), anti-SMAD3 (GeneTex, 111123, and CST, C67H9), anti-SMAD4 (CST, D3R4N), anti-MYC tag (CST, D84C12), anti-HA tag (CST, C29F4), anti-p53 (Santa Cruz, sc-126), anti-MDM2 (Millipore, OP46), and anti-β-actin (Sigma, A5441).

### Cell proliferation assay

For cell proliferation assays. 1 × 10^6^ indicated cells were plated on 10 cm dishes at day 0. Viable cells were counted on day 3 and day 6 in a hemocytometer after trypsinization and Trypan Blue staining. The results were from three biological experiments.

### Expression plasmids

The mammalian expression plasmids for Flag-tagged USP7 wildtype (WT) and CS mutant, in *pFlag-CMV2* vector, were previously described [31]. For expressing Flag-HA-tagged USP7, the cDNA of full-length *USP7* was PCR-amplified and cloned to *pIRES-Flag-HA* vector (Clontech). For the baculoviral construct, *USP7* cDNA was cloned to *pFastBac-Flag* (Invitrogen) and bacmid was prepared as described in the manufacturer’s instructions. The *pRK5-Myc-SMAD3* plasmid was a gift of Dr. Che-Chang Chang (Taipei Medical University, Taiwan). For Flag-HA-tagged SMAD3, the PCR-amplified *SMAD3* cDNA was cloned to *pIRES-Flag-HA* vector. PCR primers are listed in Supplementary Table S1. All plasmids were verified by direct DNA sequencing.

### Luciferase reporter assays

The genomic regions franking the identified *SMAD3* enhancers were PCR-amplified from the genomic DNA of H1299 cells and cloned into *pGL3-promoter* (Promega). All plasmids were verified by direct DNA sequencing. For reporter assays, 1 × 10^4^ H1299 cells were co-transfected with 50 fmol *pGL3* vectors and 2 fmol *pRL-TK*, a *Renilla* luciferase expressing vector, using Lipofectamine^TM^ 3000 according to the manufacturer’s instructions (Invitrogen). Dual-Glo luciferase assays were performed at 48 h post-transfection according to the manufacturer’s instructions (Promega). The activities of *Firefly* luciferase were normalized with the activities of *Renilla* luciferase, and results are presented as fold activity to the *pGL3-promoter* vector alone.

### ChIP-seq and ChIP-qPCR assays

The ChIP-seq and ChIP-qPCR assays were carried out as previously described [32, 33] with modifications. Briefly, H1299 cells were fixed with 1% formaldehyde, lysed in FA lysis buffer with protease inhibitor Complete cocktail. Chromatin was sonicated with a sonifier (Qsonica) followed by immunoprecipitated with anti-H3K27ac antibody (Abcam, ab4729). For ChIP-seq assays, 1 × 10^7^ cells and 3 μg antibodies were used, and isolated genomic DNAs were subjected to library construction and high-throughput DNA sequencing by the Genomic Research Center of National Yang Ming Chiao Tung University (Taiwan). Raw reads were mapped to hg19 human genome assembly, H3K27ac peaks were called by MACS software with default parameters and input DNA as control. Super-enhancer (SE) analysis was performed using the ROSE program [34] with all H3K27ac peaks identified in WT and/or *USP7* KO H1299 cells. For ChIP-qPCR assays, 3 × 10^6^ cells and 1 μg antibodies were used. Quantitative PCRs were carried out with QuantiNova Probe PCR kit (Qiagen) and the ΔΔCt method were used to determine the enrichment of indicated factors. The antibodies used for ChIP-qPCR were anti-SMAD3 (CST and C67H9), anti-SMAD4 (CST and D3R4N), and anti-USP7(Bethyl and A300-033A). All ChIP-qPCR primers are listed in Supplementary Table S1.

### RNA-seq and RT-qPCR

The RNA-seq and RT-qPCR assays were carried out as previously described [32, 33]. In brief, total RNAs were isolated using the Quick-RNA Mini-prep kit (Zymo Research). For RNA-seq analysis, total RNAs were submitted to the Genomic core of UNC at Chapel Hill for library construction and high-throughput sequencing. Raw reads were mapped to hg19 human genome assembly using the RNA STAR program in Galaxy [35]. The gene expression profilings were analyzed by HOMER software with default parameters. The RPKM of selected genes were used for generating the heatmap using the MORPHEUS software of the Broad Institute website. The gene set enrichment analysis (GSEA) was performed using the GSEA software. For RT-qPCR, gene-specific primers are listed in Supplemental Table S1.

### Co-immunoprecipitation (Co-IP) assay

HEK293T cells were co-transfected with indicated plasmids. Forty-eight hours post-transfection, cells were lysed in BC150 buffer (50 mM Tris-HCl pH 7.9, 150 mM NaCl, and 0.5 mM EDTA) containing 0.2% Triton X-100 and protease inhibitor Complete cocktail (Roche). Cleared cell lysates were immunoprecipitated with anti-HA or anti-Flag agarose beads in BC150 buffer. The immunoprecipitates were resolved by SDS-PAGE and analyzed by immunoblotting with indicated antibodies.

### CRISPR/Cas9-mediated gene editing

The *pcDNA3.3_hCas9* and gRNA_cloning plasmids [36] were obtained from Addgene (# 41815 and #41824). Guide RNAs (gRNAs) targeting exon 2 or exon 3 of *USP7* were cloned into gRNA_cloning vector according to a protocol described in the Addgene website. Cells were transiently co-transfected with *pcDNA3.3_hCas9* and gRNA-expressing vectors and selected with neomycin for 2 weeks. Knockout clones were verified by immunoblots with anti-USP7 antibody (Bethyl laboratories, A300-033A) and DNA sequencing of PCR-amplified *USP7* exon 2 or exon 3 in *pGEM-T_easy* vector.

### In vitro and in vivo deubiquitinating assay

Recombinant Flag-tagged USP7 proteins were expressed in baculovirus-infected High-Five cells and anti-Flag affinity purification was carried out as described previously [32, 33]. For isolation of mono-ubiquitinylated SMAD3 protein, HEK293 *USP7* KO cells were co-transfected with expression vectors for Flag-HA-tagged SMAD3 (*pIRES-FlagHA-SMAD3*) and myc-tagged ubiquitin (*pEF1-Myc-Ub*) using PolyJet reagent (SignaGen) according to the manufacturer’s instructions. Transfected cells were lysed in BC150 buffer supplemented with 1% Triton X-100 and a Complete cocktail. Cell lysates were immunoprecipitated with anti-Flag M2 beads (Sigma) at 4 °C for 1 h followed by extensively washed with BC150 buffer. For in vitro deubiquitinating assay, the anti-Flag immunoprecipitates were incubated with recombinant full-length USP7 WT or CS proteins at 37 °C for 1 h. The reactions were stopped by boiling in 1x Sample buffer and subjected to immunoblotting assays with indicated antibodies. For in vivo deubiquitinating assay, HEK293 WT and *USP7* KO clone were co-transfected with *pRK5-Myc-SMAD3* and *pCI-HA-Ub* vectors. Cell lysates of transfected cells were prepared in BC150 buffer supplemented with 1% Triton X-100 and Complete cocktail, and immunoprecipitated with anti-HA beads. The immunoprecipitates were analyzed by immunoblot with anti-Myc antibody.

### Xenograft mouse model

The 8–10 weeks old C57BL/6 male mice were obtained from National Laboratory Animal Breeding and Research Center (Taipei, Taiwan). For the xenograft model, mice were subcutaneously injected with 1 × 10^7^ H1299 or A549 cells suspended in a 200 μl mixture (1:1) of PBS and Matrigel (Corning). Tumor volumes were determined using the equation: 0.5 × (longest diameter) × (shortest diameter). The animal care and experimental procedures were performed in accordance with the Guidelines of the Institutional Animal Care and Use Committee (IACUC) of National Yang Ming Chiao Tung University (Taipei, Taiwan).

### Flow cytometry

For flow cytometry analyses, 2 × 10^5^ H1299 cells were incubated in media supplemented with 10% or 1% FBS for 6 days. Cell cycle profiles were analyzed by staining with 0.02 mg/mL propidium iodide (PI) solution (Sigma) containing 0.2 mg/mL RNase A (Sigma) for 30 m at room temperature. Apoptotic cells were analyzed using the Annexin V-fluorescein isothiocyanate (FITC) apoptosis detection kit (Invitrogen) according to the manufacturer’s instructions. The treated/stained cells were analyzed in a Beckman Coulter cytoFLEX cytometer.

### Statistics

All data are means ± SD from three biological experiments. Statistical analysis were performed with a one-tailed Student’s *t*-test, *P* values <0.05 were considered statistically significant.

## Results

### USP7 depletion facilitated the proliferation of p53-deficient lung cancer cells

**Fig. 1:**
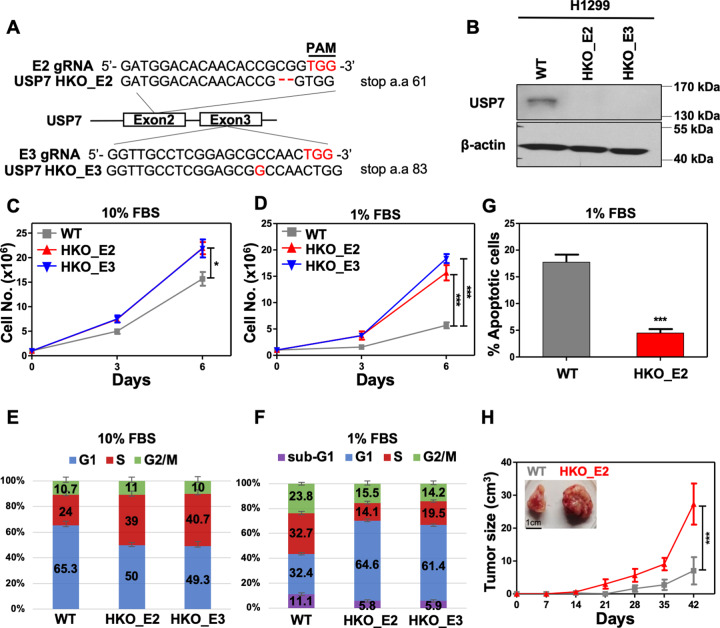
Inactivation of USP7 promotes cell progression of p53-deficient lung cancer H1299 cell line. **A** Schematic of guide RNA (gRNA) design and genomic sequences of *USP7* knockout (KO) clones. KO_E2 Exon 2 targeted, KO_E3 Exon 3 targeted, PAM protospacer adjacent motif. **B** Immunoblots of USP7 in the lysates from wildtype (WT) or indicated *USP7* KO H1299 cells. β-actin is the loading control. **C**–**F** Cell proliferation (**C**, **D**) and cell cycle profiling (**E**, **F**) assays of wildtype (WT) and *USP7* KO (HKO_E2 and HKO_E3) H1299 cell lines in culture media supplemented with 10% (**C**, **E**) or 1% fetal bovine serum (FBS) (**D**, **F**). Results are mean ± SD from three independent experiments. **G** Apoptotic cell profiling by Annexin V staining in wildtype (WT) or *USP7* KO (HKO_E2) H1299 cells treated with 1% FBS for 6 days. **H** The growth curves of xenografted tumors of wildtype (WT) or *USP7* KO (HKO_E2) H1299 cell lines. The tumor sizes were determined every 7 days using an external caliber. The data are presented as mean ± SD from a cohort of six mice. The inset showing the representative images of WT or HKO_E2 cells xenografted tumors on day 42. Student’s *t*-test, *p < 0.05; **p < 0.01; ***p < 0.001.

It is well accepted that USP7, the most studied deubiquitinating enzyme, attenuates the level of tumor suppressor p53 by protecting MDM2 and MDMX from ubiquitination-mediated proteasome degradation during cancer progression [11, 37–39]. Thus, USP7 inhibition has become a promising strategy for cancer therapy. Based on the above, USP7 inhibition-mediated tumor suppression requires a functional activity of p53; however, p53 is frequently deleted or mutated in most cancer types [40]. Therefore, it is of significant interest to understand the impact of *USP7* inactivation in p53-deficient cancer cells [12, 15, 41–44]. To address this interesting point, we performed CRISPR/Cas9-mediated gene editing to inactivate *USP7* in p53-deficient lung cancer H1299 cells. Two individual gRNAs were designed to target the exon 2 (E2 gRNA) or exon 3 (E3 gRNA) of *USP7* and Sanger sequencing analysis of selected clones revealed early stop codons at amino acid residues 61 or 83, respectively (Fig. 1A). In agreement with the genotyping, immunoblotting assays verified the knockout of endogenous *USP7* in selected H1299 clones, termed HKO_E2 and HKO_E3 (Fig. 1B). In contrast to cancer cell lines with WT p53 [40], *USP7* inactivation promoted cell progression of p53-deficient lung cancer H1299 cells (Fig. 1C) and the effect is more profound in cells treated with low serum (Fig. 1D). Interestingly, comparable levels of MDM2 were detected in both WT and USP7 KO cells (Fig. S1). These results clearly suggest that USP7 may regulate the cancer cell progression in an MDM2/p53**-**independent manner.

To investigate how USP7 attenuates the cell progression, we analyzed the cell-cycle profiles of WT and *USP7* KO H1299 cells by standard flow cytometry with PI staining. In regular culture media, we observed that *USP7* inactivation perturbs the cell-cycle profiles of H1299 cells (Fig. 1E). Notably, less sub-G1 phase cells were detected in *USP7* KO cells when treated with serum starvation (Fig. 1F). In addition, Annexin V staining assays further revealed that *USP7* inactivation significantly decreased the percentage of low-serum-induced early apoptotic cells of H1299 cells (Fig. 1G). Furthermore, the *USP7* KO H1299 cells also displayed an advanced growth in a xenograft mouse model (Fig. 1H). Although it is not clear whether the dysregulated cell-cycle profile of *USP7* KO cells has any effects on cell proliferation, our results clearly indicated that USP7 plays an important role in regulating the cell apoptosis in p53-deficient lung cancer H1299 cells in vitro and in vivo.

### USP7 is required for the function of a SE at *SMAD3* locus

**Fig. 2:**
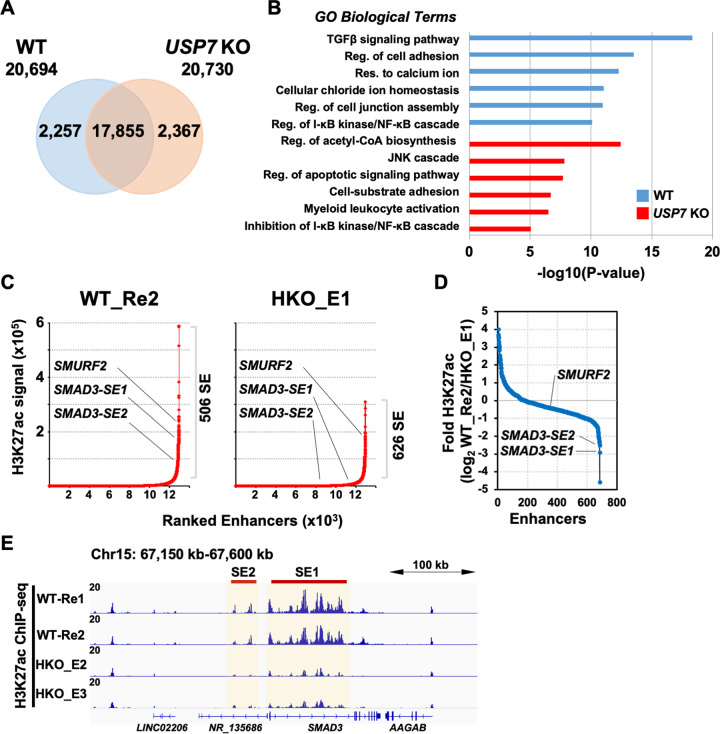
*USP7* KO selectively impairs the super-enhancer of *SMAD3*. **A** Venn diagram summarizing the H3K27ac ChIP-seq peaks identified in wildtype (WT, overlapped peaks of two biological repeats) and *USP7* KO (overlapped peaks of two independent clones HKO_E2 and HKO_E3) H1299 cells. **B** Gene ontology (GO) biological terms of *USP7* WT- and KO-specific H3K27ac ChIP-seq peaks in (**A**) using GREAT analysis. **C** H3K27ac loading across the enhancers in *USP7* WT (left) and KO (right, clone HKO_E2) H1299 cell lines. Super-enhancers (SE) were identified using the ROSE program with default parameters. Numbers of identified SE are indicated at right. The SE-associated genes involved in the TGFβ-signal pathway were denoted. **D** The plot showing the fold changes of H3K27ac ChIP-seq signals for all identified SEs in H1299 cell lines. **E** IGV gene tracks of H3K27ac ChIP-seq signals in *USP7* WT (two biological repeats) and KO (HKO_E2 and HKO_E3) H1299 cell lines at *SMAD3* locus. Identified SE domains (right, SMAD3-SE1; left, SMAD3-SE2) are yellow-colored and indicated at the top.

To identify USP7-regulated pathway/gene network that participate in apoptosis, we first analyzed the genome-wide enhancers with an antibody against acetyl-lysine 27 of Histone H3 (H3K27ac), a well-documented histone modification for transcriptionally active enhancers and promoters [45, 46], to perform chromatin immunoprecipitation followed by high-throughput sequencing (ChIP-seq) in both WT and *USP7* KO H1299 cells. Analyses of H3K27ac ChIP-seq datasets defined comparable peak numbers (~20,700) for WT (overlap of two biological repeats) and *USP7* KO (overlap of HKO_E2 and HKO_E3) (Figs. 2A, S2, and Dataset 1) cells. Interestingly, the overlapping analysis revealed that 2257 and 2367 of the H3K27ac peaks are specific for WT and *USP7* KO H1299 cells, respectively (Fig. 2A and Dataset 1). Gene Ontology (GO) analyses of these cell clone-specific H3K27ac peaks indicated that the most *USP7* KO affected GO term was the TGFβ signaling pathway in H1299 cells, whereas other identified pathways displayed weak association (Fig. 2B and Dataset 2). To further address whether these clone-specific H3K27ac peaks associate with specific enhancer clusters, the ChIP-seq datasets were subjected to SE analysis, an approach for identifying tissue/cell-specific master regulators [34]. Although generally lower H3K27ac loading scores for peaks identified in *USP7* KO cells, we identified a larger number of SEs in *USP7* KO cells than that in WT H1299 cells (Fig. 2C and Dataset 3). Interestingly, we identified two TGFβ signaling-associated genes, *SMURF2* and *SMAD3* (flanked by two SE domains), in the WT H1299 cells. Furthermore, the H3K27ac ChIP-seq signals for two *SMAD3*-associated, but not *SMURF2*-associated, SE domains were significantly downregulated in *USP7* KO H1299 cells (Fig. 2C, D). Gene tracks of H3K27ac signals verified the consistency of two USP7-dependent SE domains at *SMAD3* locus in H1299 cells (Fig. 2E). These results suggest a function of USP7 in regulating the SMAD3/TGFβ signaling pathway in H1299 cells.

### Inactivation of USP7 specifically downregulates the expression of *SMAD3*

**Fig. 3:**
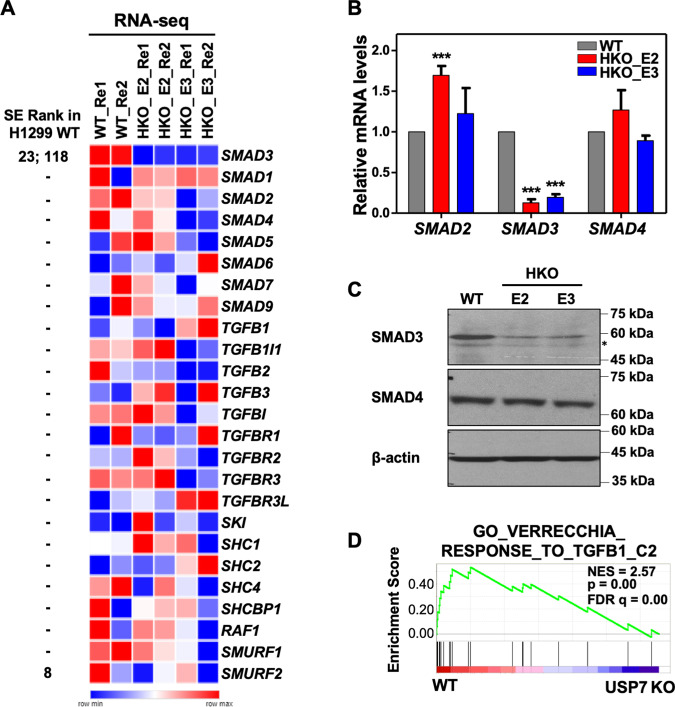
USP7 is specifically required for the expression of *SMAD3* in H1299 cells. **A** Heatmaps of RNA-seq results showing the relative expression levels of genes involved in TGFβ-signal pathway in wildtype (WT) and *USP7* KO (HKO_E2 and HKO_E3) H1299 lines. Two biological repeats for each line and rank in wildtype SE identification are shown. **B** RT-qPCR assays showing the relative expression levels of indicated genes in wildtype (WT) or *USP7* KO (HKO_E2 and HKO_E3) H1299 cell lines. Means ± SD from three biological experiments are shown. Student’s *t*-test, ***P < 0.001. **C** Immunoblots of SMAD3 and SMAD4 in the lysates of wildtype (WT) or *USP7* KO (HKO_E2 and HKO_E3) H1299 cell lines. β-actin as an internal control. Asterisk indicates a nonspecific signal. **D** GSEA plot showing the enrichment of TGFβ-responsive genes in H1299 wildtype (WT) relative to *USP7* KO (HKO_E2) cells.

Since the genome-wide profiling of enhancers and SEs indicated that USP7 may play a role in regulating the SMAD3/TGFβ signaling, further analyses were focused on the expression of genes involved in TGFβ signaling. As a complement to the enhancer profiles by H3K27ac ChIP-seq, we also performed RNA-sequencing (RNA-seq) analyses of WT and *USP7* KO H1299 cells. Notably, the downregulated expression of *SMAD3*, but not SE-associated *SMURF2* or other TGFβ signaling-associated genes, could be reproducibly observed in *USP7* KO H1299 lines (Fig. 3A and Dataset 3). The complementary quantitative RT-PCR (RT-qPCR) (Figs. 3B and S3) and immunoblotting (Fig. 3C) assays further confirmed the specific USP7 requirement for the optimal expression, but not protein stability (Fig. S4), of the *SMAD3*. Although we didn’t identify many dysregulated genes, GSEA analysis of our RNA-seq results denoted that TGFβ signaling was enriched in WT H1299 (Fig. 3D and Dataset 3), which agrees with identified GO terms of enhancer profiles (Fig. 2B). With these results, we conclude that USP7 is specifically required for the optimal expression of *SMAD3* in H1299 cells.

### USP7 is implicated in the *SMAD3* autoregulatory loop

**Fig. 4:**
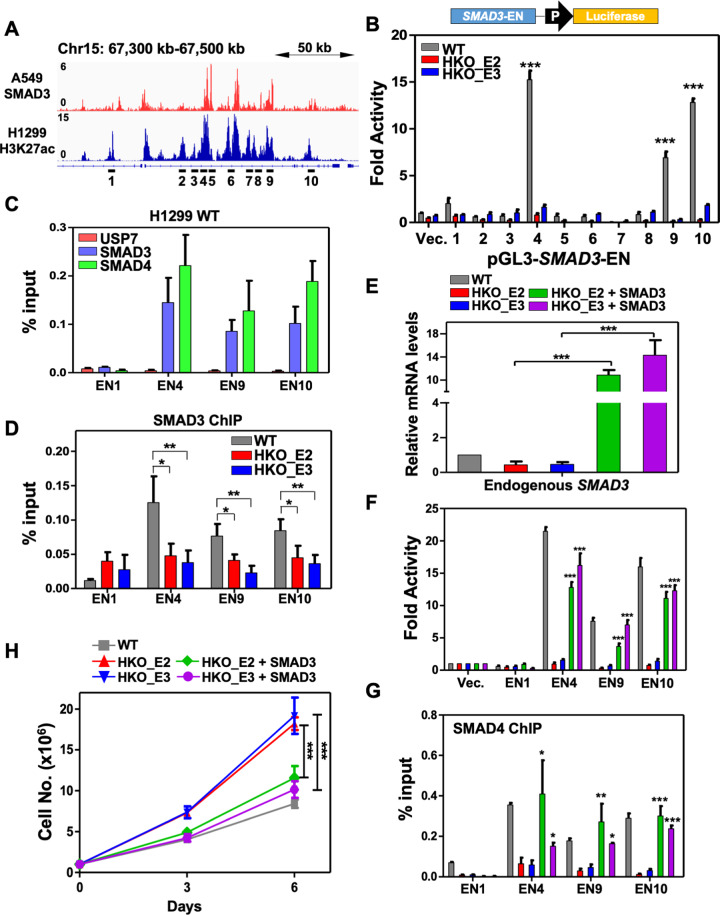
USP7 is required for the positive autoregulation of *SMAD3*. **A** IGV gene tracks at *SMAD3* locus showing the ChIP-seq profiles of SMAD3 binding in A549 line (GSE51509) and H3K27ac occupancies in the H1299 line. The constituent enhancers within the identified SE domains are numbered and denoted at the bottom. **B** Luciferase reporter assays showing the relative enhancer activities of the *SMAD3* SE constituents driven the expression of a mini-SV40 promoter-reporter (upper) in H1299 wildtype (WT) and *USP7* KO cell lines. Fold activities relative to *pGL3*-promoter (Vec.) are shown as means ± SD, triplicated experiments. **C** ChIP-qPCR assays showing the binding of USP7, SMAD3, and SMAD4 at indicated *SMAD3* SE constituents in H1299 wildtype (WT) cells. Note, the detection of USP7 binding at these regions was failed. **D** ChIP-qPCR assays showing the binding of SMAD3 at indicated SMAD3 SE constituents, as described in (**B**), in H1299 wildtype or *USP7* KO cell lines. **E** RT-qPCR assays showing the relative expression levels of endogenous *SMAD3* in H1299 wildtype (WT) and *USP7* KO cells without or with ectopically expressed SMAD3. Same cell treatments were used for panels **E** to **G**. **F** Luciferase reporter assays showing the relative enhancer activities of indicated SMAD3 SE constituents in indicated cell treatments. **G** ChIP-qPCR assays showing the binding of SMAD4 at indicated *SMAD3* SE constituents in indicated cell treatments. **H** Cell proliferation assays showing the growth of H1299 wildtype or *USP7* KO cells without or with ectopically expressed SMAD3. Throughout the figure, mean ± SD from three independent experiments. Student’s *t*-test, *p < .05; **p < .01; ***p < .001.

Our above results indicated that USP7 is important for the function of two SEs at the *SMAD3* locus and for the expression of *SMAD3*. To further identify the SE constituents regulated by USP7, we carried out the reporter assays with luciferase constructs driven by the genomic regions spanning the identified H3K27ac peaks (EN1 to EN10), except the peak within the core promoter, at *SMAD3* locus (Fig. 4A). Interestingly, we also noticed that the regions of identified H3K27ac peaks in H1299 cells highly coincide to the SMAD3 ChIP-seq peaks in the lung cancer A549 cell line, suggesting a common *SMAD3* autoregulatory loop [47]. Correspondingly, the luciferase reporter assays revealed that three H3K27ac-occupied genomic regions (EN4, EN9, and EN10) displayed robust enhancer activities in H1299 cells, and, notably, their activities were completely abolished in *USP7* KO lines (Fig. 4B), indicating an essential role of USP7 for the function of these SE constituents. Since we failed to detect the USP7 binding to these three genomic regions (Fig. 4C), and since SMAD3 and SMAD4 coincidently binds to these USP7-responsive enhancer regions (Fig. 4C), further analyses focused on the *SMAD3* autoregulatory function on these SE constituents. In agreement with the above results, ChIP-qPCR assays showed an USP7-dependent chromatin binding of SMAD3 to the EN4, EN9, and EN10, but not the EN1, sites (Fig. 4D). Given that USP7 is required for the expression of *SMAD3*, it is possible that the reduced SMAD3 binding at these enhancers was a reflection of downregulated SMAD3 level in *USP7* KO lines. To further verify the autoregulatory function of SMAD3 on these SE constituents, we ectopically expressed SMAD3 in *USP7* KO lines. As expected, the SMAD3 restoration enhanced the expression of endogenous *SMAD3* (detected by primers at 3′UTR of *SMAD3*) (Fig. 4E) and rescued the activities of selected SE constituents in reporter assays in *USP7* KO lines (Fig. 4F). To exclude the effect of ectopic expression, the binding of SMAD3-SMAD4 heterodimer to the selected SE constituents was monitored by SMAD4 ChIP assay. Consistent with the above observations, the binding of SMAD4 to the selected SE constituents was also impaired in *USP7* KO lines and rescued in SMAD3-expressed *USP7* KO lines (Fig. 4G). Furthermore, the SMAD3 restoration also reversed the *USP7* KO-enhanced cell proliferation (Fig. 4H). Taken together, our results suggest that *USP7* KO impairs the SMAD3 autoregulation and leads to abnormal cell proliferation.

### Deubiquitinase function is required for USP7-mediated activation of *SMAD3*

**Fig. 5:**
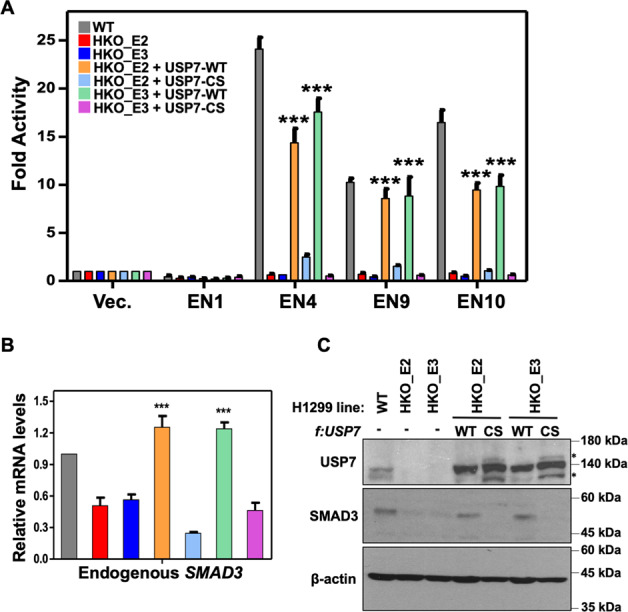
The deubiquitinase activity of USP7 is required for USP7-mediated transcriptional activation of *SMAD3*. **A** Luciferase reporter assays showing the relative enhancer activities of *SMAD3* SE constituents in H1299 wildtype and *USP7* KO cells without or with ectopically expressed USP7 wildtype (WT) or deubiquitinase-deficient CS mutant. **B**, **C** The relative mRNA (**B**) or protein (**C**) levels of endogenous SMAD3 were determined by RT-qPCR or immunoblotting assays, respectively, in H1299 cells described in (**A**). Mean ± SD from three independent experiments. Student’s *t*-test, ***p < .001. Asterisks indicate nonspecific signals.

To investigate whether the deubiquitinase function of USP7 is involved in the activation of *SMAD3*, *USP7* KO H1299 lines were transduced with expression constructs for USP7 WT or deubiquitinase-deficient CS mutant forms and subjected to the following assays. Notably, luciferase reporter assays indicated that the functions of selected SE constituents were recovered by the ectopic USP7 WT, but not CS mutant, in *USP7* KO lines (Fig. 5A). Furthermore, the mRNA (Fig. 5B) and protein (Fig. 5C) levels of endogenous *SMAD3* were also restored by ectopic USP7 WT, but not CS mutant. These results establish a deubiquitinase dependency in regulating the USP7-mediated activation, in particular the positive autoregulation, of *SMAD3* in H1299 cells.

**Fig. 6:**
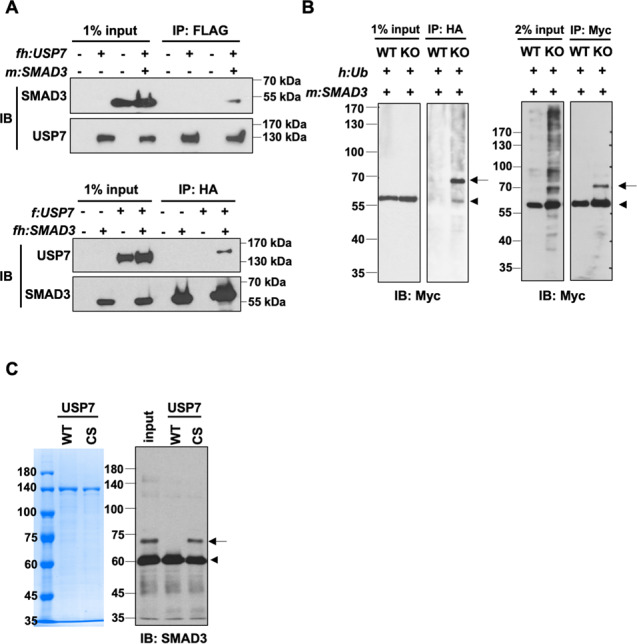
USP7 interacts with SMAD3 and deubiquitinates the mono-ubiquitinated SMAD3. **A** Co-immunoprecipitation (co-IP) assays with anti-Flag (upper) or anti-HA (bottom) resins showing the interactions between USP7 and SMAD3 in HEK293T cells transfected with indicated expression constructs. fh Flag-HA, m Myc, f Flag. **B** Immunoprecipitation (IP) assays showing SMAD3 is mono-ubiquitinated in the absence of USP7. HEK293 wildtype (WT) or *USP7* KO (KO) cells were co-transfected with expression vectors for HA-tagged ubiquitin (h:Ub) and Myc-tagged SMAD3 (m:SMAD3). IPs were performed with anti-Myc and anti-HA antibodies followed by immunoblotting with anti-Myc antibody. Arrow and arrowhead indicate mono-ubiquitinated and unmodified SMAD3, respectively. **C** Immunoblots of SMAD3 (right) in the reaction product of in vitro deubiquitinating assays showing the removal of mono-ubiquitin from SMAD3 by recombinant USP7 wildtype (WT) but not deubiquitinase-deficient CS mutant. Mono-ubiquitinated SMAD3 was isolated from transfected HEK293 cells as described in (**B**), recombinant USP7 proteins (left) were purified from baculovirus-infected insect cells.

It was previously reported that SMAD3 is mono-ubiquitinated and this modification blocks the DNA-binding function of SMAD3 by disrupting its interaction with SMAD4 [30]. Since USP7 is one of the most active deubiquitinases and our above results indicated the enzymatic function of USP7 is required for the activation of *SMAD3*, we hypothesized that USP7 may directly regulate the deubiquitination of SMAD3. To address this point, we first inspected the interaction between SMAD3 and USP7. As shown in Fig. 6A, reciprocal co-immunoprecipitation assays confirmed an interaction between ectopic USP7 and SMAD3 in HEK293T cells. To examine whether USP7 is involved in deubiquitination of SMAD3, indicated HEK293 cells were cotransduced with expression constructs for HA-tagged ubiquitin and Myc-tagged SMAD3 followed by anti-HA and anti-Myc immunoprecipitations. Notably, a slower migrating band of SMAD3 (by anti-Myc immunoblot) was detected only in the immunoprecipitates from the lysates of *USP7* KO cells (Fig. 6B)—confirming the role of USP7 in catalyzing the de-monoubiquitination of SMAD3 in cells. To further assess the direct function of USP7 in the removal of mono-ubiquitin from SMAD3, mono-ubiquitinated SMAD3 proteins were isolated from *USP7* KO HEK293 cells ectopically expressed HA-Ub and Myc-SMAD3 (Fig. 6B) and subjected to in vitro deubiquitination with highly purified recombinant USP7 (Fig. 6C, left panel). The results showed that conjugated mono-ubiquitin on SMAD3 was efficiently removed by USP7 WT, but not the CS mutant, proteins (Fig. 6C). Collectively, our results suggest that USP7 interacts with and catalyzes the removal of the repressive mono-ubiquitin from SMAD3 protein.

### USP7 inactivation decreased cell progression of p53-positive but enhanced that of p53-negative, lung cancer A549 cell line

**Fig. 7:**
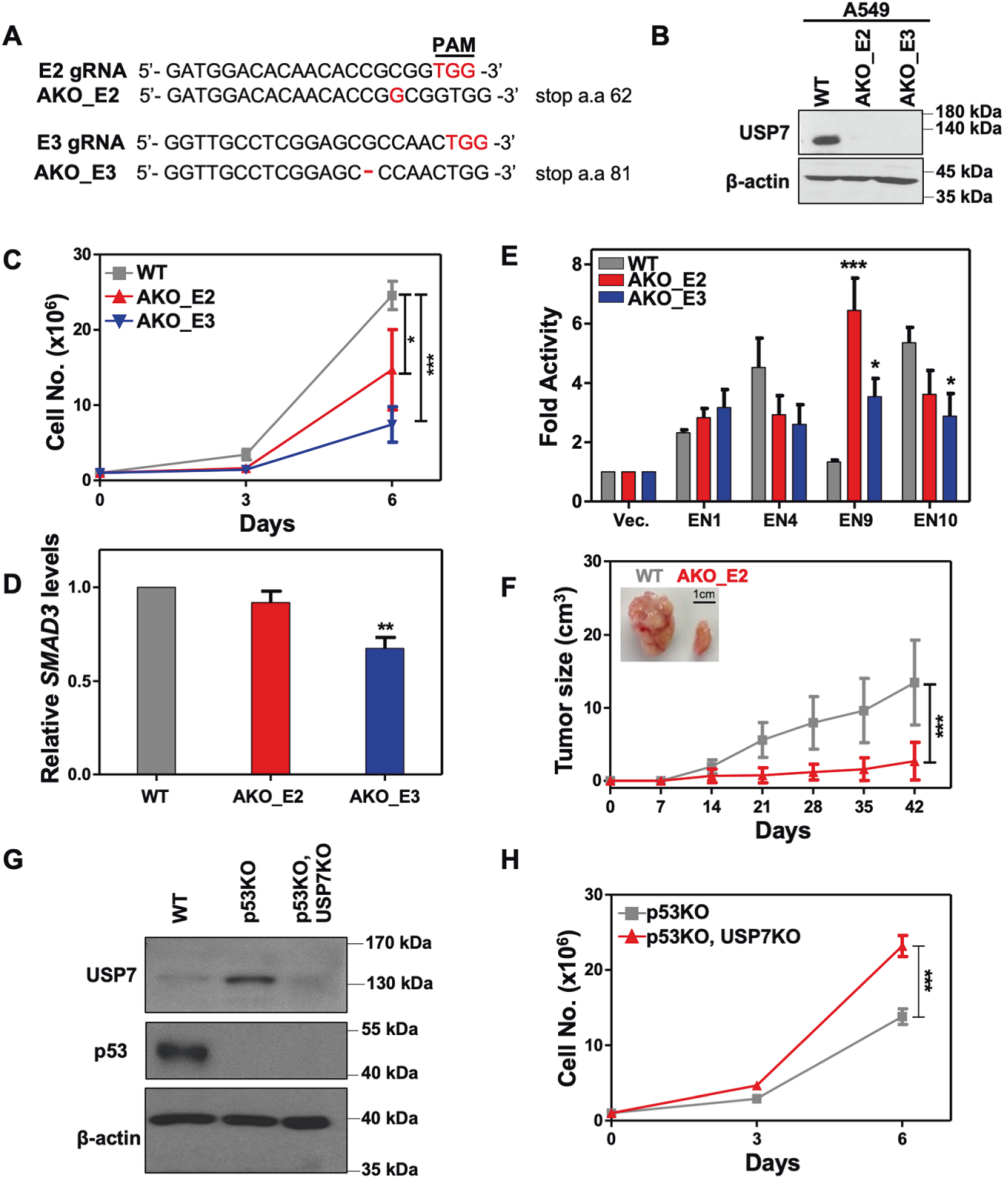
Inactivation of USP7 decreases cell progression of p53-positive lung cancer A549 cell line. **A** Schematic of guide RNA (gRNA) design and genomic sequences of *USP7* knockout (KO) A549 clones. AKO_E2 Exon 2 targeted; AKO_E3 Exon 3 targeted. **B** Immunoblots of USP7 in the lysates of wildtype or *USP7* KO A549 cells. β-actin is the loading control. **C** Cell proliferation assays showing the growth of wildtype (WT) and *USP7* KO A549 (AKO_E2 and AKO_E3) cell lines. Data were means ± SD from three independent experiments. **D** RT-qPCR assays showing the *SMAD3* mRNA levels in wildtype (WT) or *USP7* KO A547 cell lines. **E** Luciferase reporter assays showing the relative enhancer activities of indicated *SMAD3* SE constituents in wildtype (WT) and *USP7* KO A549 cells. The normalized luciferase activity of pGL3-promoter was set to 1. **F** The growth curves of xenografted tumors with wildtype (WT) or *USP7* KO (AKO_E2) A549 cell lines. The tumor sizes were determined every 7 days using an external caliber. The data were presented as means ± SD from a cohort of six mice. The inset showing the representative images of tumors on day 42. Student’s *t*-test, *p < 0.05; **p < 0.01; ***p < 0.001. **G** Immunoblots of USP7 and p53 in the lysates of wildtype (WT), *p53* KO, and *p53* and *USP7* double KO A549 cells. β-actin as an internal control. **H** Cell proliferation assays showing the growth of *p53* KO or *p53* and *USP7* double KO cell lines. Data were means ± SD from three independent experiments. Student’s *t*-test, ***p < 0.001.

Although many previous reports showed that inactivation of USP7 using inhibitory compounds or RNAi-mediated gene silencing hampered the growth of cancer cells, our results clearly demonstrated that *USP7* KO impaired the *SMAD3* autoregulation and resulted in enhanced growth of p53-null H1299 cells. We propose that the opposite effect of *USP7* inactivation in these studies may due to the activity of the p53/MDM2 pathway, the major targeted signaling in USP7 inactivation-mediated tumor suppression. To address this hypothesis, we have attempted to express p53 in WT and *USP7* KO H1299 lines and, however, ectopic expression of p53 potently inhibited the growth of both lines (data not shown). Alternatively, we inactivated *USP7* in the p53-positive lung cancer A549 line. Direct DNA sequencing of indicated exons (Fig. 7A) and immunoblot assays (Fig. 7B) confirmed the successful knockout of *USP7* in isolated A549 clones. In contrast to the enhanced growth of the *USP7* KO H1299 line (Fig. 1C, D), decreased cell proliferation was observed in *USP7* KO A549 lines (Fig. 7C). Furthermore, RT-qPCR assays of *SMAD3* (Fig. 7D) and luciferase reporter assays of selected SMAD3 SE constituents (Fig. 7E) demonstrated that *USP7* inactivation may not or weakly affected the activation of *SMAD3* in A549 cells, probably due to non-detectable monoubiquitination of SMAD3 in this A549 line (data not shown). In agreement with previous reports of tumor suppression by targeting USP7, inactivation of *USP7* also impaired the tumor progression of A549 cells in the xenograft mouse model (Fig. 7F). Finally, to further validate our hypothesis, we inactivated the p53 in WT and *USP7* KO A549 lines (Fig. 7G) and observed that *USP7* inactivation enhanced the cell progression of p53-negative A549 lines in regular culture media (Fig. 7H). Collectively, these results suggest that USP7 targeting may serve as a strategy for tumor suppression in p53-positive, but not p53-negative, lung cancer cells.

## Discussion

**Fig. 8:**
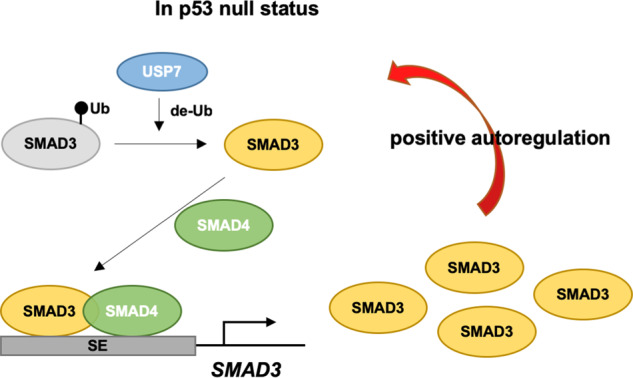
Model for USP7 mediates the positive autoregulation of *SMAD3* in p53-deficient lung cancer cells. Based on previous and our current results, the following working model for USP7-mediated autoregulation of SMAD3 is proposed: (i) the monoubiquitination of SMAD3 inhibits its interaction with coregulatory SMAD4 that impairs their DNA-binding function, (ii) the deubiquitinase function of USP7 directly catalyzes the removal of mono-ubiquitin from SMAD3, and (iii) the unmodified SMAD3 forms a heterodimer with SMAD4, binds to multiple SE constituents of SMAD3 locus, and enhances the expression of *SMAD3*, (iv) upregulated SMAD3 facilitates its positive autoregulation and controls the cell cycle progression.

USP7 is one of the well-studied deubiquitinases and is involved in multiple cellular pathways, including tumorigenesis. Previous studies have demonstrated that RNAi-mediated silencing or small compound-mediated inhibition of USP7 resulted in destabilizing the MDM2/MDMX proteins that leading to activate the p53-mediated tumor suppression, thus USP7 can serve as a promising target for cancer treatment. Given the tumor suppression function of p53, accumulated reports have indicated that *TP53* is one of the most mutated genes in various tumors [40]. It is of great interest for characterizing the impact of USP7 inactivation in p53-deficient tumors. Toward this goal, we employed the CRISPR/Cas9-mediated gene editing to inactivate the USP7 in p53-null lung cancer H1299 cells and analyzed the changes of gene activation and expression. We found that USP7 inactivation facilitated cell proliferation and tumor growth of p53-deficient lung cancer cells, which may due to the diminished autoregulatory loop of SMAD3, a key regulator of TGFβ signaling in regulating both apoptosis and cancer metastasis. In addition, we also found that USP7 is required for the SMAD3 positive autoregulation by catalyzing the deconjugation of repressive mono-ubiquitin from SMAD3 that could promote the establishment of SE at SMAD3 locus by the SMAD3-SMAD4 heterodimer. Base on previous and our current findings, we present a previously unappreciated model for tumor modulator of USP7 through its roles as a de-monoubiquitinase for SMAD3 in p53-deficient lung cancer cells (Fig. 8).

### The repressive function of USP7 in cancer cell progression

Accumulated studies have revealed that USP7 is highly upregulated in various tumors, including prostate, breast, ovarian, and colorectal cancers, as well as multiple myeloma and gliomas [10, 48]. In these tumors, a general function of USP7 in promoting the cancer cell progression was proposed to regulate the MDM2/MDMX-p53 pathway. Although USP7 was reported to interact with both MDM2/MDMX and p53, the USP7 binding by MDM2 is much stronger than by p53 [49]. Therefore, USP7-mediated deubiquitination and stabilization of MDM2/MDMX results in p53 turnover and leads to cancer cell progression [11, 38]. In addition to MDM2/MDMX-p53, USP7 was also reported to mediate the deubiquitination of other regulators to promote the metastasis and aggressiveness of tumors, including b-catenin [50], estrogen receptor [51], and mediator of DNA damage checkpoint protein 1 (MDC1) [52]. Conversely, it was also reported that USP7 is negatively associated with the prognosis of adenocarcinomas of non-small lung cancer (NSCLC) [9] and the progression of the human colorectal xenograft model [12]. However, the underlying mechanism for the repressive function of USP7 in cancer cell progression was not clearly investigated. Using H1299 cells, a p53-null NSCLC line, we unexpectedly observed that knockout of USP7 induces cell proliferation and inhibits serum-starvation-induced apoptosis. Surprisingly, however, gene-wide profilings (active enhancers and transcriptome) showed no significant changes of cell cycle or apoptotic pathways but, instead, revealed the downregulation of TGFβ signaling and impaired SMAD3 autoregulatory loop. Depending on the states of cancers, TGFβ/SMAD3 signaling can act as an oncogenic factor promoting the tumor cell invasion and metastasis in advanced cancers or functions as a regulator to inhibit cell proliferation and induce apoptosis in early-phase tumors. Although further analyses are required to determine the underlying mechanism for TGFβ/SMAD3 signaling-induced apoptosis in NSCLC, our results established an interplay between USP7 and TGFβ/SMAD3 signaling in regulating cancer cell progression in p53-null NSCLC cells.

### The deubiquitinase activity of USP7 regulates the SMAD3 function

Given the robust deubiquitinase activity and a broad range of protein substrates, USP7 has been implicated in multiple cellular pathways by mainly mediating the Lys-48 linked deubiquitination and stabilization of various kay regulators, including the abovementioned MDM2/MDMX-p53, EBNA1, ICP0, DAXX, LSD1, MDC1, and CHK1 [10, 48]. Here we show that USP7 catalyzes the deconjugation of mono-ubiquitin from SMAD3 that facilitates its interaction with SMAD3 and has no effect on its protein stability. Consistent with our observation, the USP7 was also reported to mediate the removal of mono-ubiquitin from tumor suppressor PTEN [53] and transcription factor FOXO4 [54] that triggers their nuclear exclusion. In addition, a recent study indicated that hypoxia-induced K63-polyubiquitinated USP7 can also act as a transcriptional scaffold to regulate gene transcription through direct interaction with chromatin-bound HIF −1α and facilitate the CBP recruitment to enhance the downstream gene expression [31]. Although USP7 directly interacts with SMAD3, our ChIP-qPCR assay revealed no USP7 chromatin association to the identified SMAD3 SE constituents. This observation suggests that USP7 may merely act as a deubiquitinating enzyme that erase the repressive mono-ubiquitin of SMAD3 to maintain the activity of the SMAD3 autoregulatory loop. Further analyses may be required to determine this USP7-SMAD/TGFβ pathway in other cancer types.

## Supplementary information

SupplementaryData

Dataset 1

Dataset 2

Dataset 3

## Data Availability

ChIP-seq and RNA-seq data have been deposited in the NCBI Gene Expression Omnibus database (accession number GSE172506).
