# Supplementary material for: USP7 facilitates SMAD3 autoregulation to repress cancer progression in p53-deficient lung cancer
Source: Cell Death Dis. 2021 Sep 27;12(10):880. doi: 10.1038/s41419-021-04176-8 (PMC8476631; doi:10.1038/s41419-021-04176-8)

Supplementary Data for

USP7 Facilitates SMAD3 Autoregulation to Repress Cancer Progression in p53-deficient Lung Cancer

Yu-Ting Huang[1], An-Chieh Cheng[1], Hui-Chi Tang[2], Guo-Cheng Huang[1], Ling Cai[3,4], Ta-Hsien Lin[1,5], Kou-Juey Wu[6], Ping-Hui Tseng[1], Greg G. Wang[3,4], Wei-Yi Chen[1,7,*]

[1]Institute of Biochemistry and Molecular Biology, National Yang Ming Chiao Tung University, Taipei 112, Taiwan

[2]Department of Life Sciences and Institute of Genome Sciences, National Yang Ming Chiao Tung University, Taipei 112, Taiwan

[3]Department of Biochemistry and Biophysics, University of North Carolina at Chapel Hill School of Medicine, Chapel Hill, NC, USA

[4]Lineberger Comprehensive Cancer Center, University of North Carolina at Chapel Hill School of Medicine, Chapel Hill, NC, USA

[5]Basic Research Division, Medical Research Department, Taipei Veterans General Hospital, Taipei 112, Taiwan

[6]Cancer Genome Research Center, Chang Gung Memorial Hospital at Linkou, Taoyuan, Taiwan

[7]Cancer Progression Research Center, National Yang Ming Chiao Tung University, Taipei 112, Taiwan

*Correspondence:

Wei-Yi Chen

Institute of Biochemistry and Molecular Biology, National Yang Ming Chiao Tung University, Taipei 112, Taiwan

Contact: chenwy@nycu.edu.tw

Phone: +886-2-2826-7328

Supplemental Figures

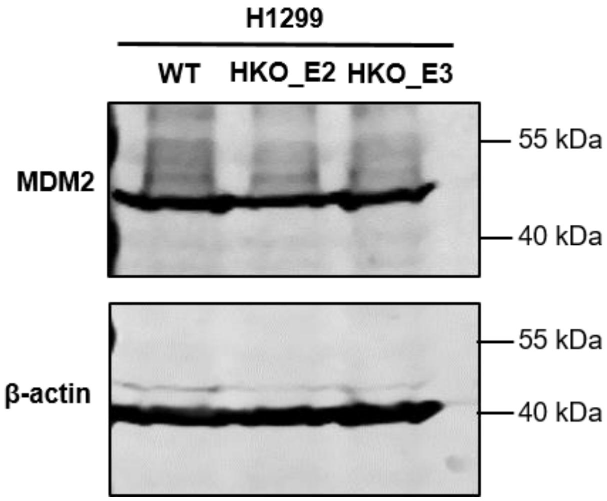

Figure S1. Comparable levels of MDM2 are expressed in wildtype and *USP7* KO H1299 cells.

Immunoblots of MDM2 in the cell lysates from wildtype (WT) and the indicated *USP7* KO H1299 cells. β-actin is the loading control.

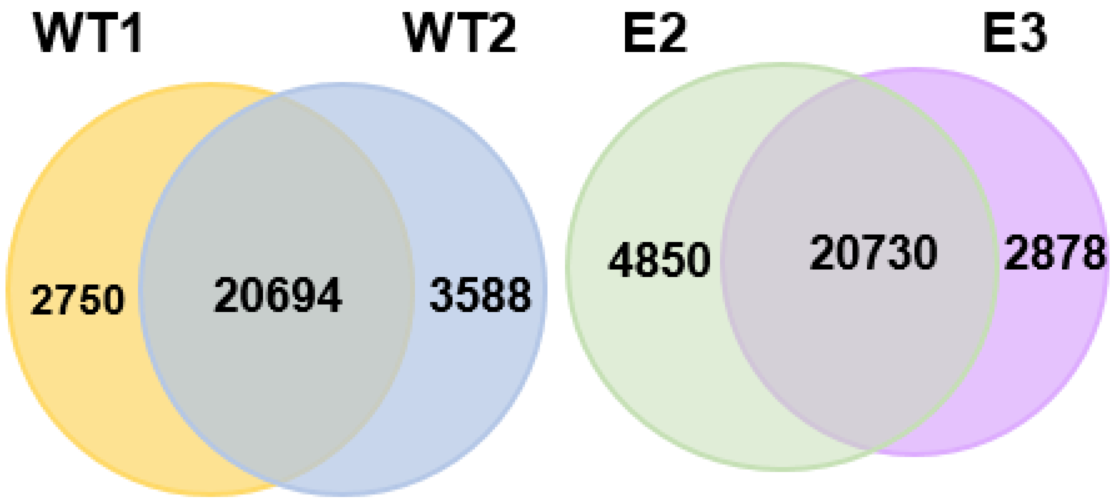

Figure S2. Genome-wide profiling of USP7-responsive enhancers in H1299 cells.

Venn diagrams showing the comparison of H3K27ac ChIP-seq peaks identified in wildtype (left panel, two biological experiments) or in *USP7* KO (right panel, two clonal lines: HKO-E2 and HKO-E3) H1299 lines. Peak overlapping analysis was performed using the *mergePeaks* of HOMER software with "-d given" parameter. The common regions of each genomic background were further analyzed for identifying the USP7-responsive enhancers in Fig. 2A and listed in dataset S1.

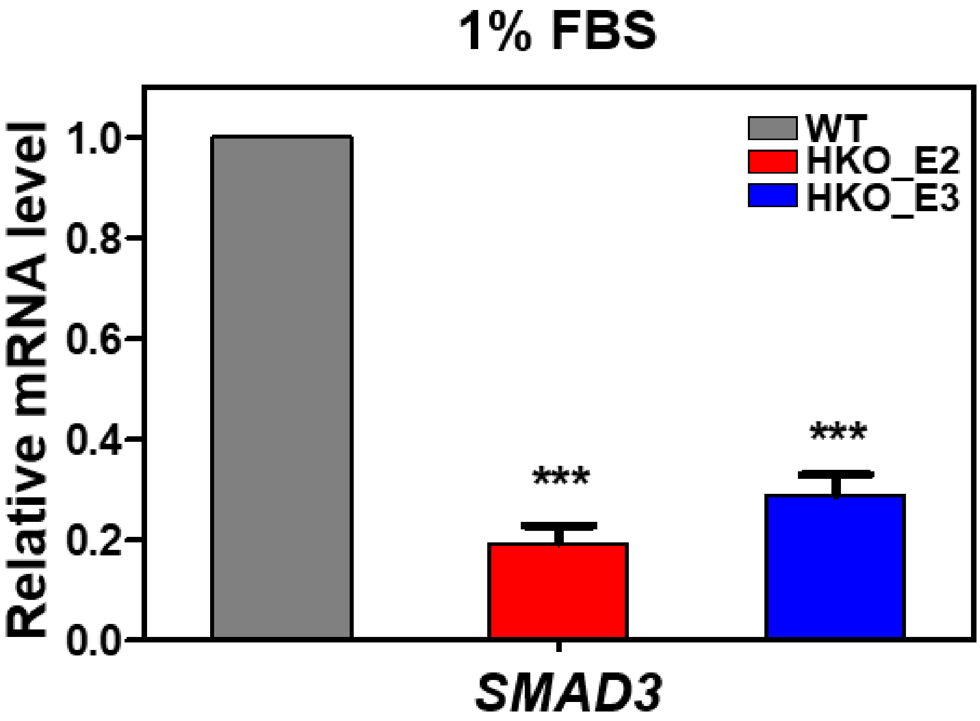

Figure S3. USP7 inactivation significantly downregulates the expression of SMAD3.

RT-qPCR assays showing the relative expression levels of *SMAD3* in wildtype (WT) or *USP7* KO (two independent clones: HKO_E2 and HKO_E3) H1299 cell lines treated with 1% FBS. Means ± SD from three biological experiments are shown. Student's t-test, *** $p < 0.001$.

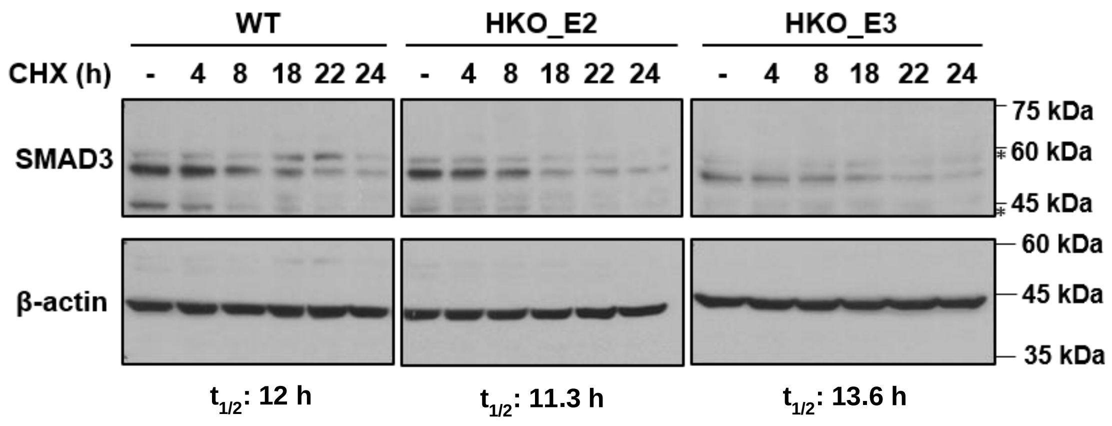

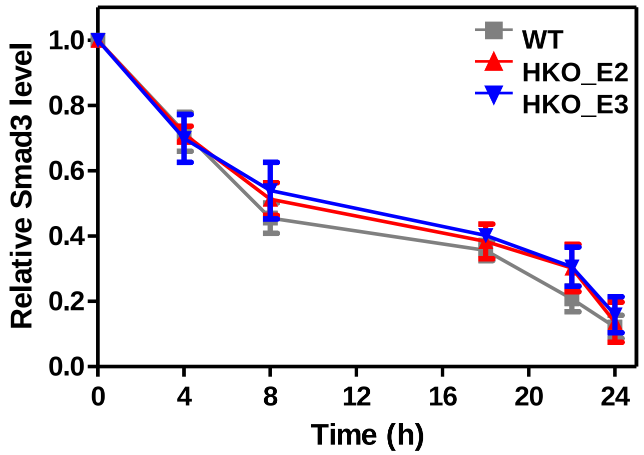

Figure S4. USP7 inactivation has no effects on the SMAD3 protein stability. (Upper) Representative immunoblots showing the stability of SMAD3 in wildtype or *USP7* KO H1299 lines. Cells were treated with cycloheximide (CHX, 20 μg/mL) for indicated time (h) and cell lysates were subjected to immunoblotting assays with anti-SMAD3 antibody. β-actin as an internal control. Asterisks denote non-specific signals. Calculated half-lives ($t_{1/2}$) of SMAD3 in each cell line are indicated.

(Bottom) Decay curves for SMAD3 protein in the indicated cell lines. The SMAD3 levels were quantitated by the ImageJ software and the level in the control cells (without CHX treatment) was set to 1. Mean ± SD from 3 independent experiments.

Supplemental Table S1. Oligos used in this study

RT-qPCR

SMAD2	F: GCTGGCCTGATCTTCACAGT
	R: CCAGAGGCGGAAGTTCTGTT
SMAD3	F: GCTGTCTACCAGTTGACCCG
	R: AGGACCTTGTCAAGCCACTG
SMAD4	F: GGACTGCACCATACACACCT
	R: AATGGGAGGCTGGAATGCAA

ChIP-qPCR

SMAD3_EN1	F: AACTGCTCCAGAAACTCTCAA
	R: CACATGAAGCCCAAACCTGTG
SMAD3_EN4	F: TCTCTCTATCGCCAACGTGA
	R: TCCTGGCAGGCCTTTCCTTA
SMAD3_EN9	F: GTTGCTTTCGCCTAACTGGC
	R: AGCAAAGGGATCCACAGACG
SMAD3_EN10	F: GGAAGCAGAGTGGTATTCAGCA
	R: TAGGCAACATGGGGAAAATGGA

For reporter construction

SMAD3_EN1	F: CACATGCTATCTTCACAGTGTGATCGA
	R: GACAGAAAAAGAAAATAATGTTGACTTCAGTTTGCA
SMAD3_EN2	F: GAATCCTGGTTTTCCAAGTGTTTAGAGG
	R: GATCAGGAGGCCTCCAGCAG
SMAD3_EN3	F: TGTGTGCTTGCTCTGAAGATTCCA
	R: TTGCCTCTGTGCTGCCAAG
SMAD3_EN4	F: TTGCCTCTGTGCTGCCAAG
	R: AAAATGATTGCTTCCTGAGGTCTGGATG
SMAD3_EN5	F: GGTCTCCCCTTAAATGTCATCTAAGAGAG
	R: CGCGGGAGGTGGTGG
SMAD3_EN6	F: CTGTTCCCCCCAGACCCTG
	R: ATAGCAAGACCTCTTCTCAACAGAAAAATACAAAAA
SMAD3_EN7	F: AAATCAAGGAACATTGCCCCATCTCC
	R: CTCGGTAAGCACCAGCACATCT

SMAD3_EN8	F: CTTGGCCTGTTGGTGGTGG
	R: GAAAGAATCCAACAACTCAGATATGCAAAATTTTACC
SMAD3_EN9	F: CAGTAATTCTGCAGCCTCCCTCAC
	R: CCAGTCCCAGCTGAGATTCAGA
SMAD3_EN10	F: TCCATAGATCTGACTCTGGAAACACCG
	R: CCTGCCTGTGATTTCACAAGTGT

Fig. 1B

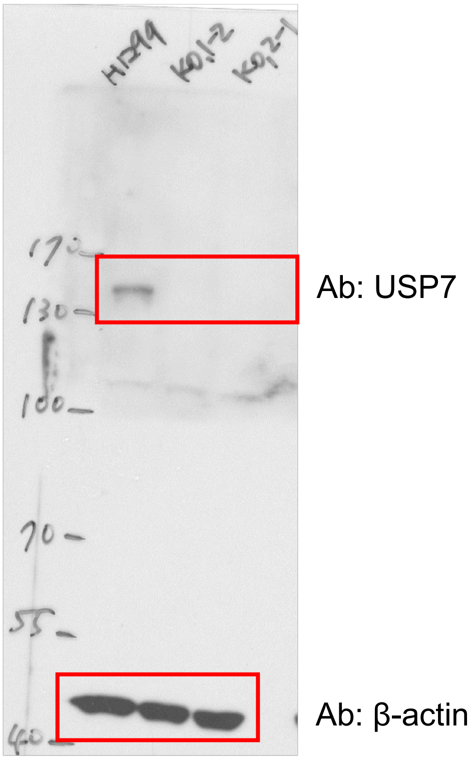

Fig. 3C

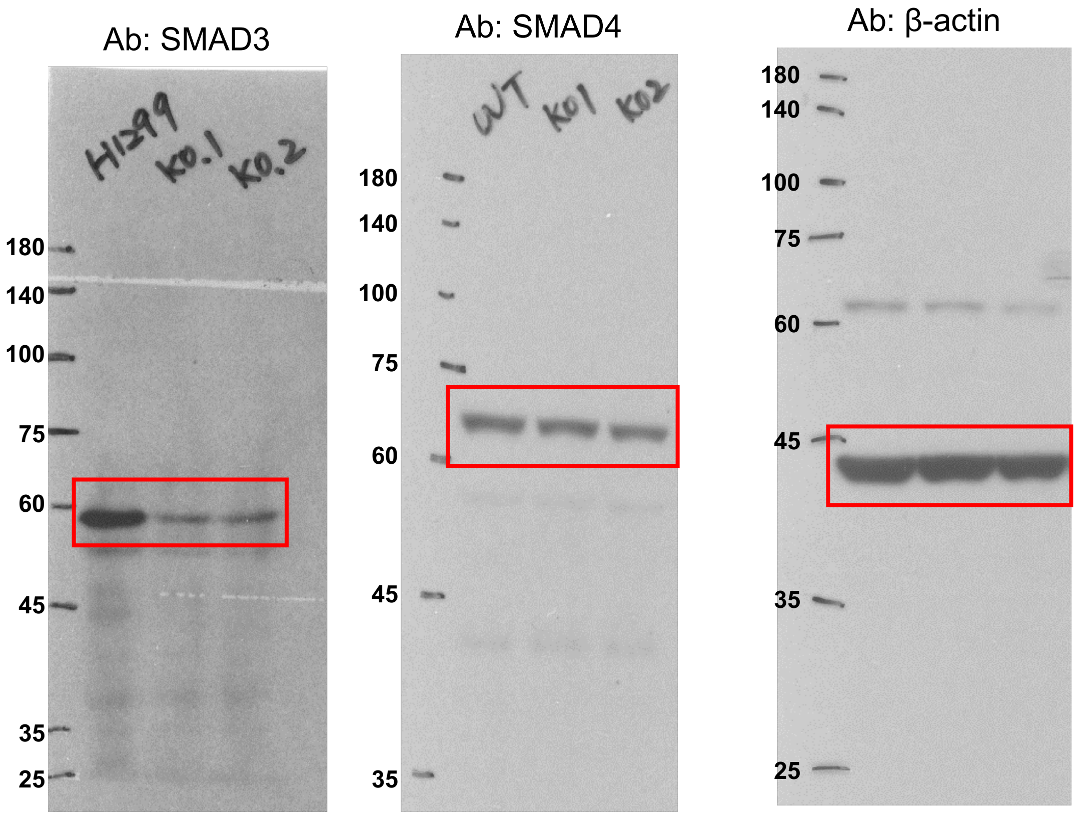

Ab: SMAD3
H1299
KO.1
KO.2
180
140
100
75
60
45
35
25
Ab: SMAD4
WT
KO1
KO2
180
140
100
75
60
45
35
Ab: β-actin
180
140
100
75
60
45
35
25

Fig. 5C

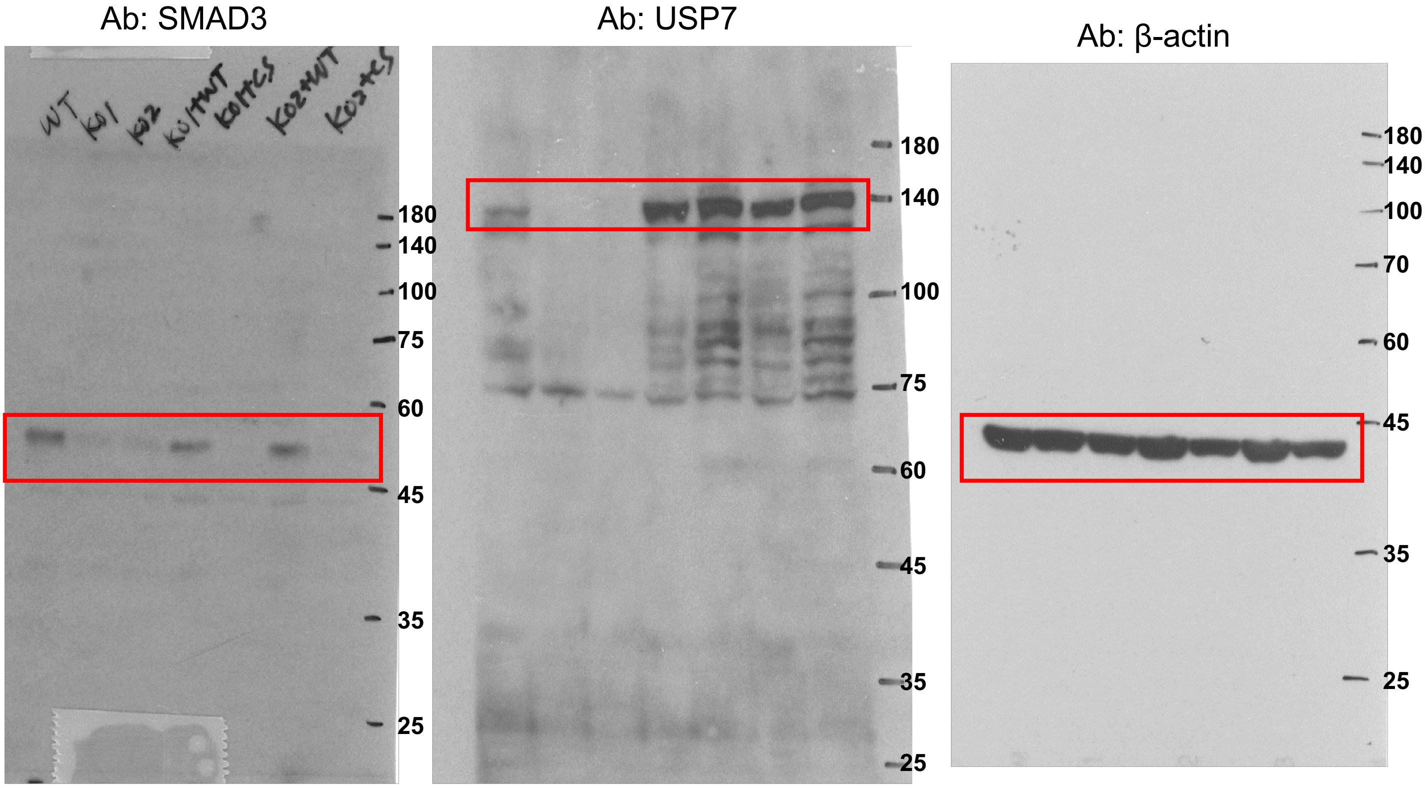
Ab: SMAD3
Ab: USP7
Ab: β-actin
WT
KO1
KO2
KO1+WT
KO1+CS
KO2+WT
KO2+CS
180
140
100
75
60
45
35
25
180
140
100
75
60
45
35
25
180
140
100
70
60
45
35
25

Fig. 6A

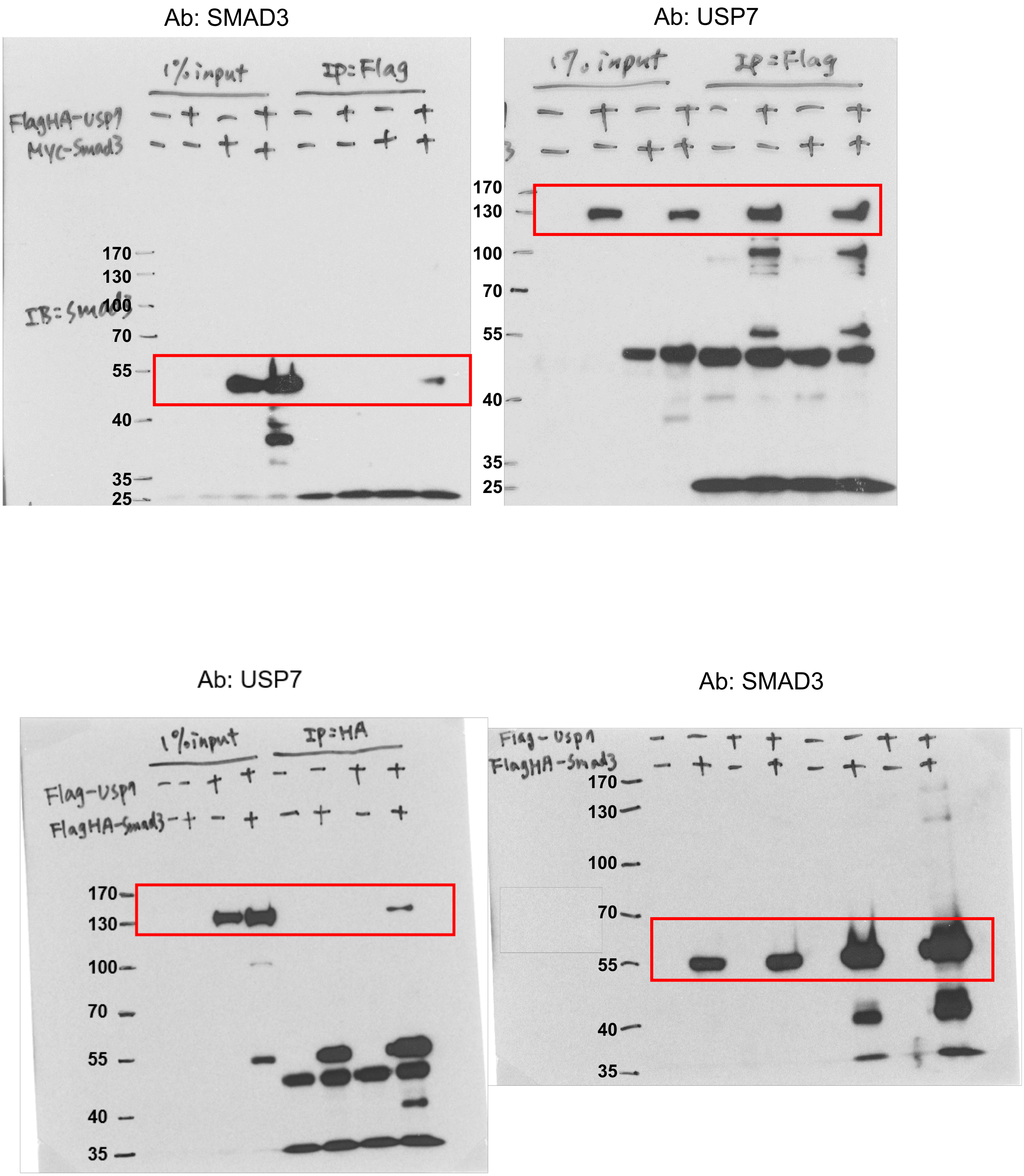
Ab: SMAD3
1% input
IP: Flag
FlagHA-USP7
Myc-Smad3
170
130
100
70
55
40
35
25
Ab: USP7
1% input
IP: Flag
170
130
100
70
55
40
35
25
Ab: USP7
1% input
IP: HA
Flag-USP7
FlagHA-Smad3
170
130
100
70
55
40
35
Ab: SMAD3
Flag-USP7
FlagHA-Smad3
170
130
100
70
55
40
35

Fig. 6B

Ab: Myc

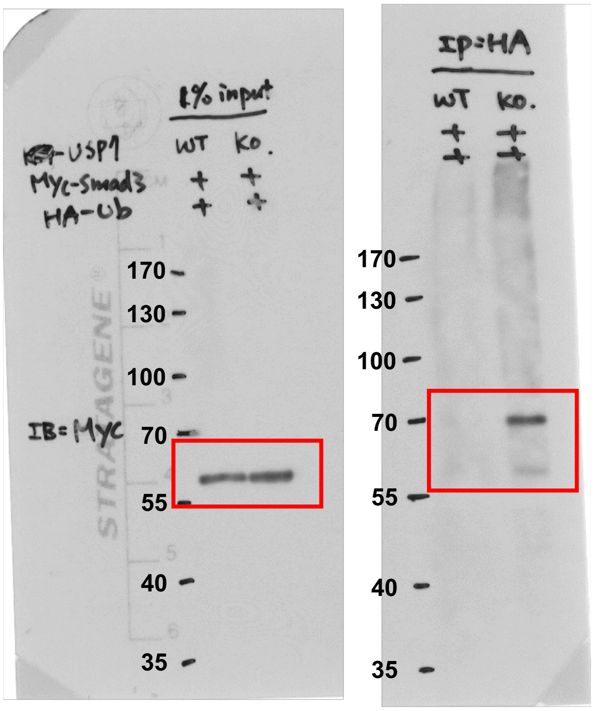
1% input
WT
KO.
-USP7
Myc-Smad3
HA-Ub
IB=Myc
170
130
100
70
55
40
35
Ip:HA
WT
KO.
170
130
100
70
55
40
35

Fig. 6C

Ab: SMAD3

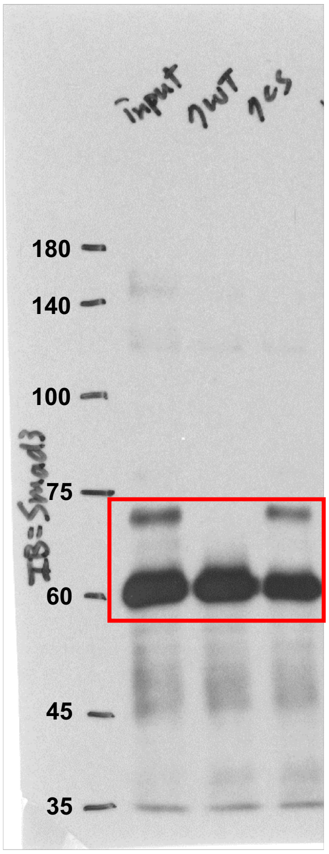

Fig. 7B

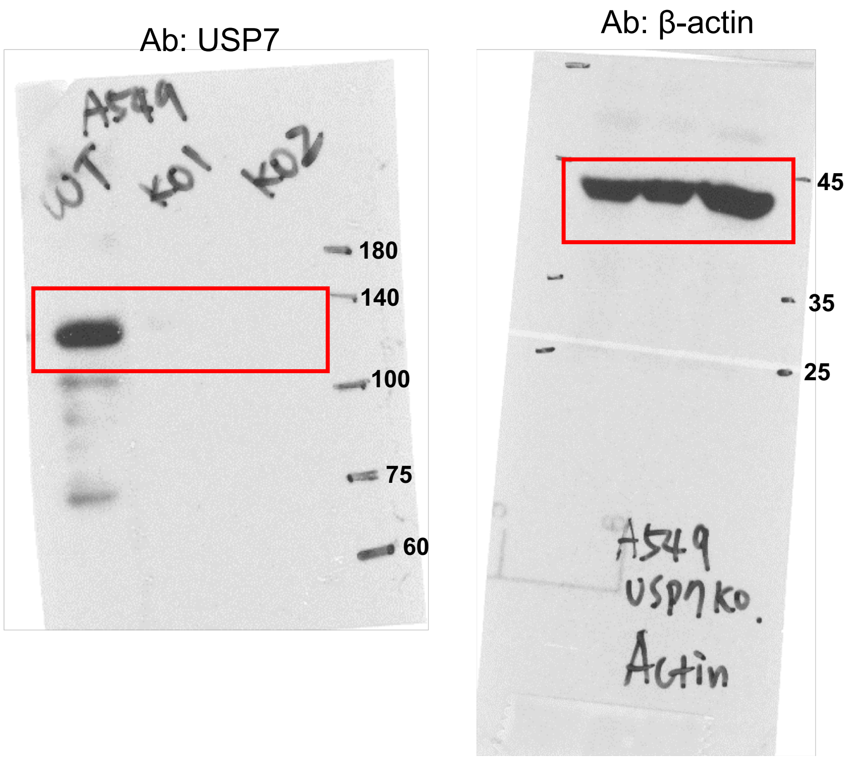

Ab: USP7
A549
WT
KO1
KO2
180
140
100
75
60
Ab: β-actin
45
35
25
A549
USP7KO.
Actin

Fig. 7G

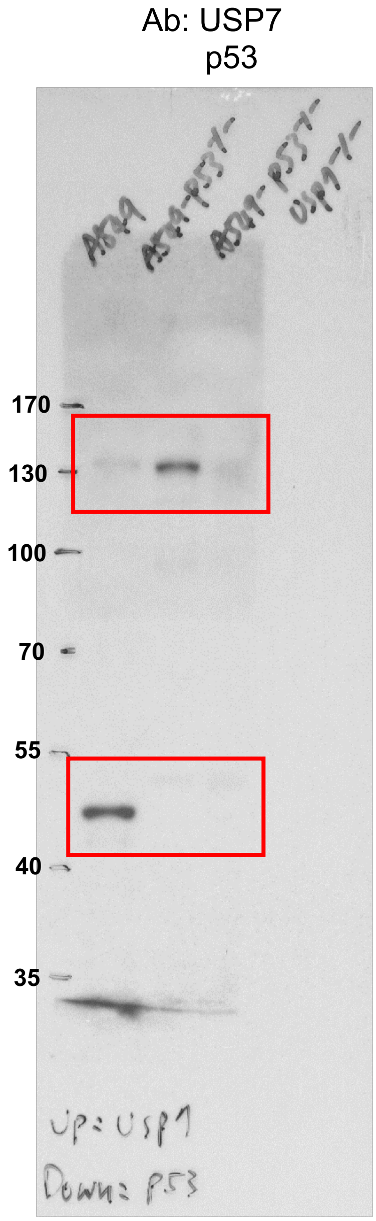
Ab: USP7
p53
170
130
100
70
55
40
35
A549
A549-p53-/-
A549-p53-/- USP7-/-
Up: USP7
Down: p53

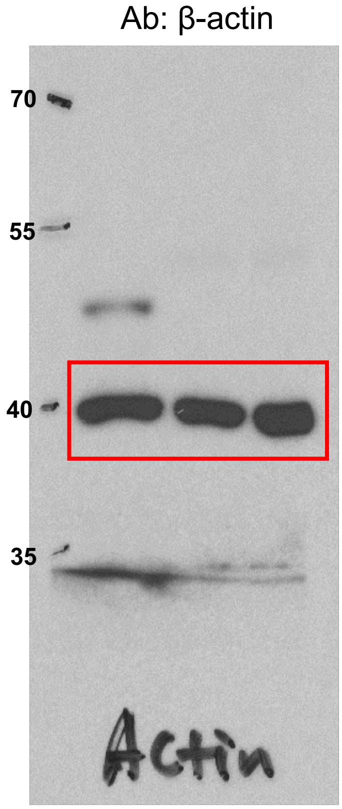
Ab: β-actin
70
55
40
35
Actin

Figure S1

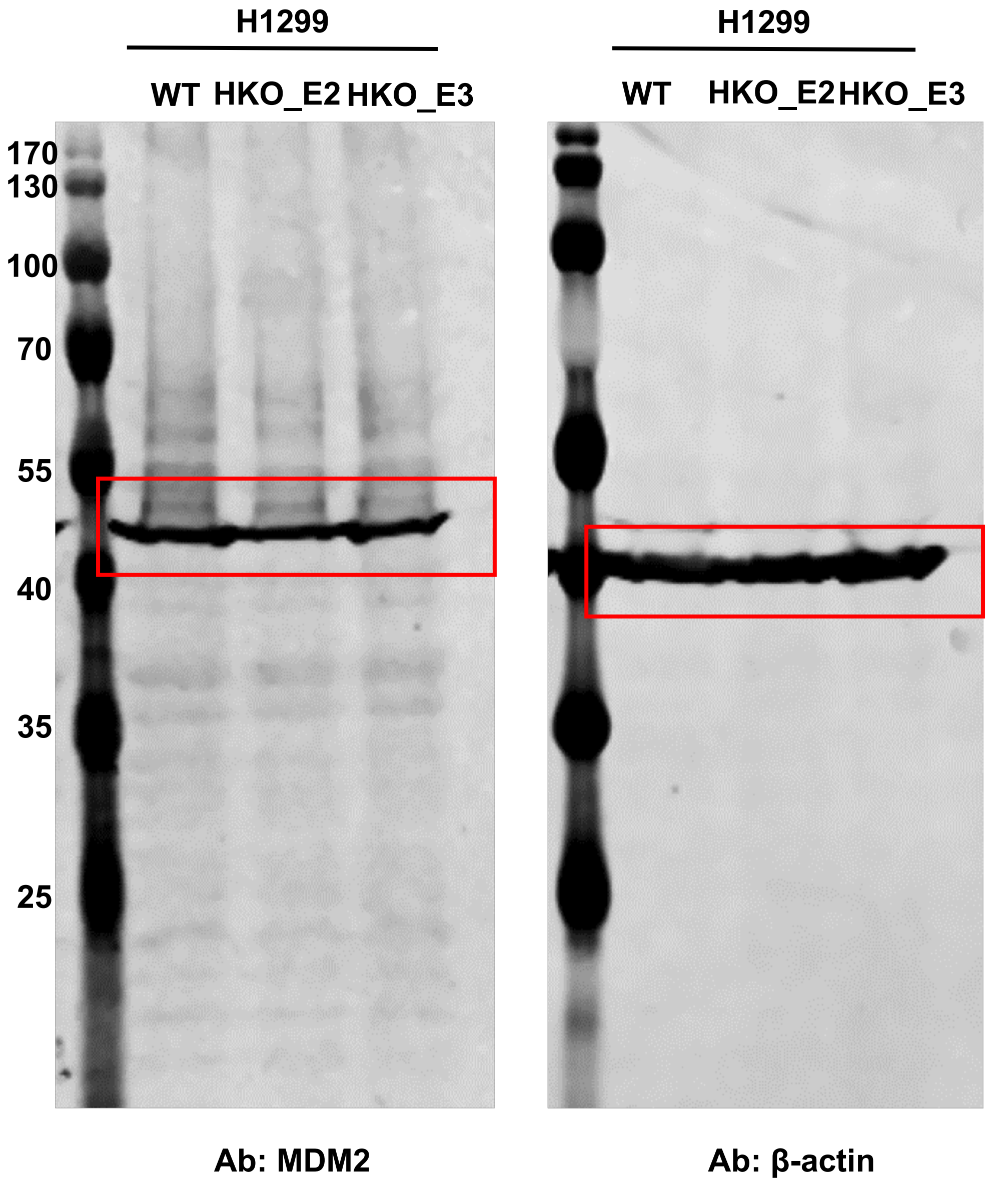

H1299
WT
HKO_E2
HKO_E3
170
130
100
70
55
40
35
25
Ab: MDM2
H1299
WT
HKO_E2
HKO_E3
Ab: β-actin

Figure S3

Ab: SMAD3

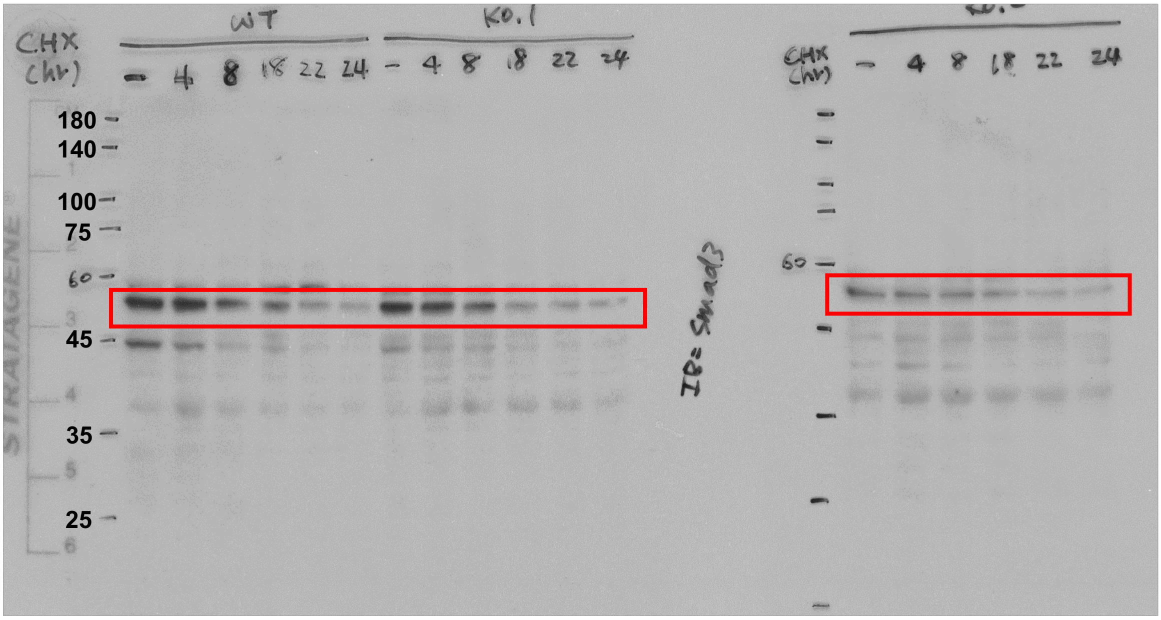

Ab: β-actin

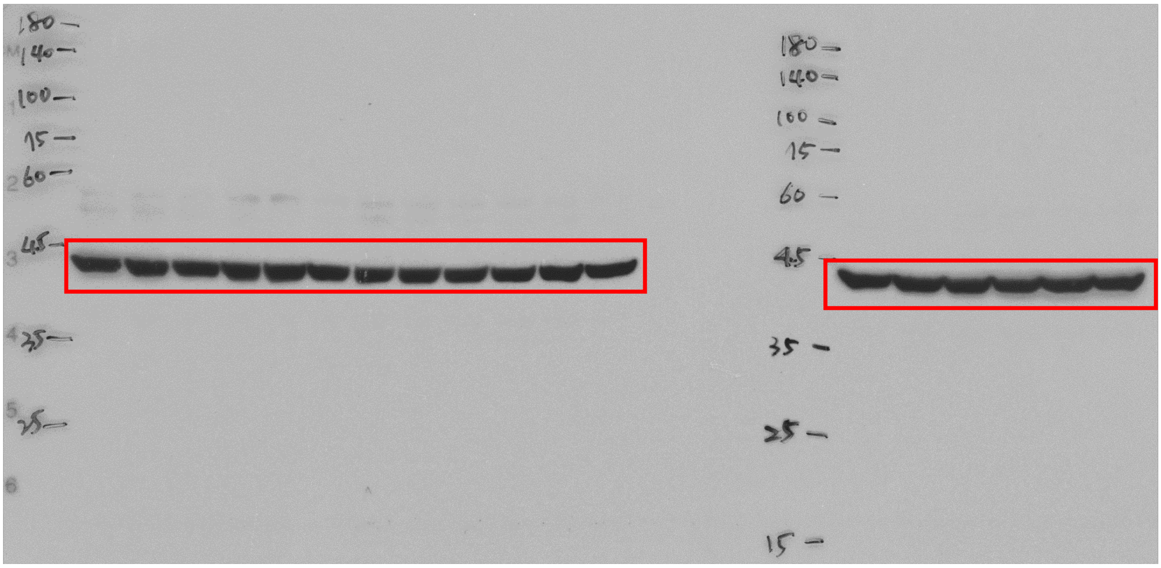

Supplement: Supplementary file 1 — SupplementaryData [file 41419_2021_4176_MOESM1_ESM.pdf]
